# Early post-contrast T1 mapping yields maximal discriminatory capacity for detection of cardiac amyloid - influence of temporal T1 differences on MOLLI imaging

**DOI:** 10.1186/1532-429X-16-S1-P322

**Published:** 2014-01-16

**Authors:** Mitchell A Cooper, Thanh Nguyen, Mathew Maurer, Heather Landau, Jiwon Kim, Sattar Gojraty, Silvina P Dutruel, Martin Prince, Yi Wang, Jonathan W Weinsaft

**Affiliations:** 1 Radiology, Weill Cornell Medical College, New York, New York, USA; 2 Cardiology, Weill Cornell Medical College, New York, New York, USA; 3 Biomedical Engineering, Cornell University, New York, New York, USA; 4 Oncology, Memorial Sloan Kettering Cancer Center, New York, New York, USA; 5 Cardiology, Columbia University Medical Center, New York, New York, USA; 6 Cardiology, Memorial Sloan Kettering Cancer Center, New York, New York, USA

## Background

Myocardial T1 mapping is increasingly used to diagnose and quantify disease burden in patients with known or suspected cardiac amyloid. Optimal timing for diagnostic application of T1 mapping is not established.

## Methods

Myocardial T1 mapping was performed in two cohorts - (1) "amyloid +" subjects, defined by biopsy-proven systemic amyloid with associated remodeling suggestive of cardiac involvement (left ventricular [LV] hypertrophy and/or atrial dilation); (2) normative controls without risk factors for amyloid or cardiovascular disease. CMR (1.5T) included 2 components - cine-CMR (SSFP) for cardiac structure/function, and T1 mapping for myocardial tissue characterization. T1 mapping was performed using a conventional modified look locker inversion recovery (MOLLI) sequence (flip angle = 30°; matrix 256 × 128; parallel imaging reduction factor =1.5; linear view ordering; 6 Kaiser-Bessel ramp preparation; 17 heart beat acquisition), with T1 calculated using an established formula (T1 = T1* (B/A-1), T1*, A, and B obtained via three-parameter exponential fit). To test time dependent differences in myocardial T1, MOLLI was acquired at sequential time points (3,5,10,14, 20 minutes) following intravenous administration of gadolinium (0.2 mmol/kg).

## Results

**Figure 1:**
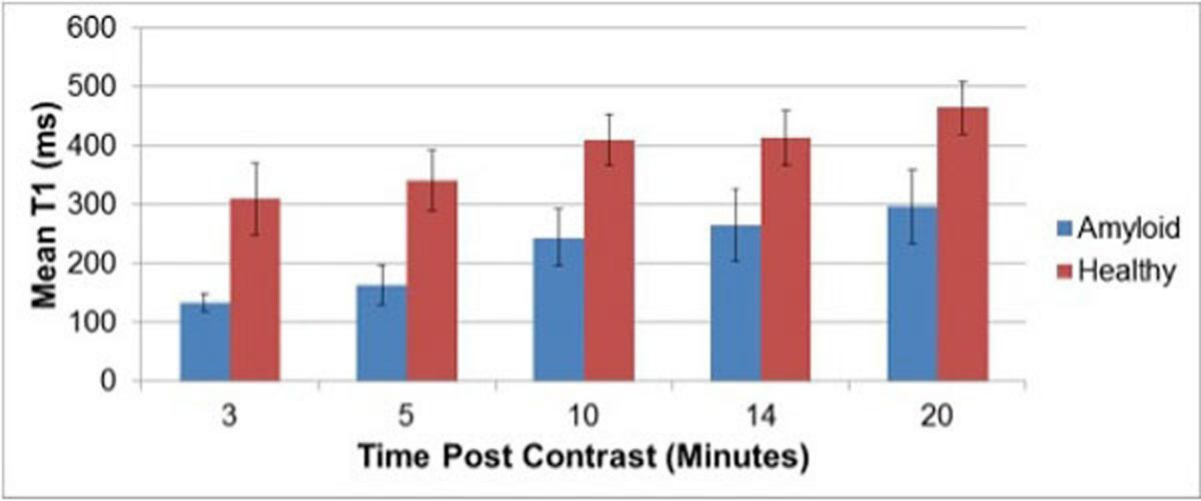
**Mean +/- standard deviation T1 times for healthy controls and amyloid patients**.

**Figure 2:**
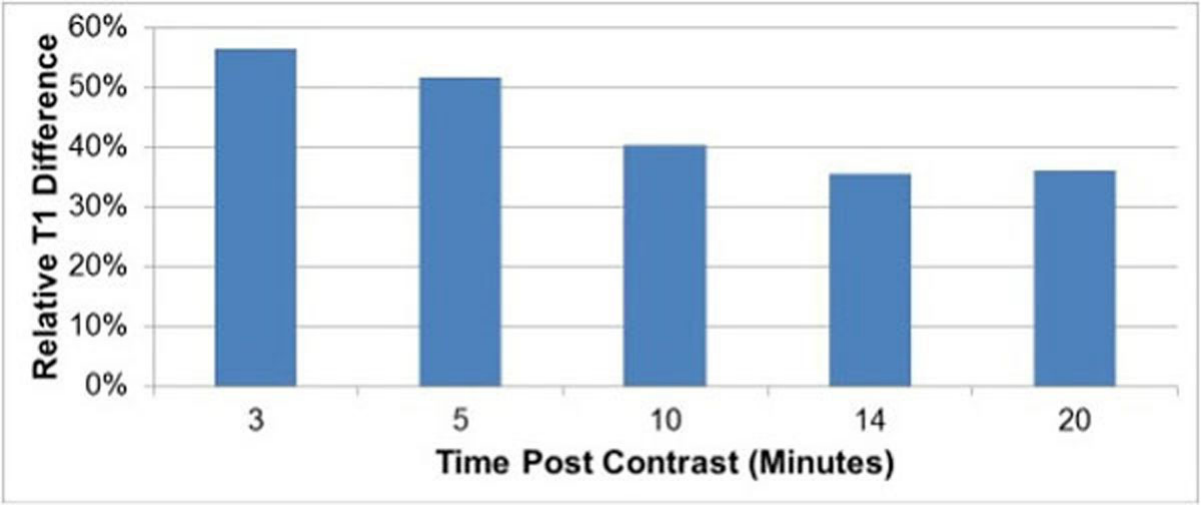
**Percentage T1 difference relative to the healthy mean T1 time**.

10 subjects (5 amyloid, 5 controls) were studied (44 ± 21 years, 40% male); all amyloid affected subjects had biopsy-confirmed disease (4 light chain type, 1 transthyretin). Amyloid subjects had higher LV mass (200 ± 34 vs. 101 ± 34 gm, p = 0.004), lower myocardial contraction fraction (32 ± 8 vs. 88 ± 27, p = 0.002), and larger left atrial area (26 ± 5 vs. 18 ± 5 cm2, p = 0.03) than controls, but similar LV end-diastolic volume (120 ± 24 vs. 133 ± 45 ml, p = 0.61), stroke volume (66 ± 10 vs. 86 ± 30 ml, p = 0.20), and LVEF (56 ± 10 vs. 65 ± 4%, p = 0.11). MOLLI was successfully acquired in all subjects at each time point: T1 differed significantly (all p ≤ 0.05) between amyloid and control groups at all times (Figure 1). However, magnitude of difference temporally decreased following gadolinium administration (Figure 2): T1 differences between patients and controls were maximal at 3 minutes post-contrast (135 ± 15 vs. 310 ± 61 msec, p = 0.004) with progressive decrements thereafter, as evidenced by 57% relative difference between groups at 3 minutes and only a 36% difference at 20 minutes following gadolinium infusion.

## Conclusions

MOLLI-quantified myocardial T1 yields maximal difference between amyloid-affected subjects and normative controls within 3 minutes following gadolinium administration. Increased magnitude of T1 difference early post contrast may relate to altered gadolinium contrast kinetics due to amyloid-associated increases in cardiac extracellular volume, and/or pulse-sequence aspects of the MOLLI approach. Current findings support use of early post-contrast MOLLI T1 mapping for identification of cardiac amyloid.

## Funding

M. Cooper was supported by NSF GFRP DGE-0707428. J. Weinsaft was supported by NIH K23HL102249-01.

